# Clinical trial of dental implants in titanium-zirconium alloy compared to titanium

**DOI:** 10.6026/973206300222224

**Published:** 2026-04-30

**Authors:** Mohamed Amir Khan, Devarshi Pandya, Koguru Madhavi, Ratna Rachel Ponraj, Santanu Mukherjee, Md Kafeel Ahmed, Rubeena Naaz

**Affiliations:** 1 Department of Oral and Maxillofacial Surgery, Registrar Oral and Maxillofacial Surgeon, Buraydah, Kingdom of Saudi Arabia; 2 Department of Periodontology, Government Dental College and Hospital, Jamnagar, Gujarat, India; 3 Department of Periodontology and Implantology, Consultant, Shine N Smile Advanced Dental Specialty, Hyderabad, Telangana, India; 4 Department of Orthodontics and Dentofacial Orthopaedics, Manipal University College Malaysia, Melaka, Malaysia; 5 Department of Dentistry, JMN Medical College and Research, Chakdaha, Nadia, West Bengal, India; 6 Department of Periodontology and Implantology, MNR Dental College and Hospital, Sangareddy, Hyderabad, Telangana, India; 7 Dental Surgeon, SRK Dental Clinic, Hyderabad, Telangana, India

**Keywords:** Dental implants, Titanium-zirconium Alloy (Ti-Zr), osseointegration

## Abstract

Titanium dental implants remain the gold standard for oral rehabilitation due to their biocompatibility and osseointegration. Therefore, it is of interest to enhance mechanical strength and stability in low-bone-volume cases where traditional implants may underperform. This randomized clinical trial compared Ti-Zr alloy implants versus conventional pure titanium implants in patients requiring prosthetic rehabilitation, evaluating primary stability, marginal bone loss, peri-implant inflammation and survival rates at regular intervals. Both implant types demonstrated comparable osseointegration rates and overall survival, with standardized clinical assessments conducted throughout the observation period. Ti-Zr implants exhibited superior mechanical stability and better peri-implant bone preservation, particularly in compromised low-density bone sites. Thus, we show Ti-Zr alloy implants as a reliable alternative to conventional titanium, especially for challenging cases requiring enhanced strength and reduced diameters.

## Background:

Dental implants are the treatment of choice for replacing missing teeth because they can restore function, aesthetics and quality of life. Notably, the use of commercially pure titanium as the gold-standard material in endosseous dental implants has been discussed since the groundbreaking work of Per-Ingvar Brånemark, owing to its high biocompatibility, corrosion resistance and capacity to achieve predictable osseointegration [1]. In most studies, titanium has a stable oxide layer on its surface, which enhances direct bone-to-implant contact, contributing to long-term clinical success rates of up to 90% [2, 3]. Despite these benefits, certain medical scenarios pose biomechanical challenges for traditional titanium implants. A decrease in bone volume, a decrease in the cross-sectional area of ridges and the need to use small-diameter implants can weaken implants and increase the risk of fracture or mechanical failure [4]. Also, when the occlusal load is high, implants with smaller diameters have higher crestal bone loss or lower survival rates [5]. The restrictions have prompted an investigation into other implant materials that can provide enhanced mechanical properties while remaining biologically compatible.

Improved development in implant biomaterials has led to titanium-zirconium (Ti-Zr) alloys for implants. Addition of zirconium to titanium forms a solid-solution alloy that enhances tensile strength and fatigue resistance, while maintaining corrosion resistance and the potential for integration into bone [6]. It has been experimentally proven that Ti-Zr alloys have higher mechanical strength than commercially pure titanium and are therefore especially appropriate for small-diameter implants and regions with limited bone support [7]. Surface alterations of Ti-Zr implants have also been shown to induce desirable cellular behaviors, such as osteoblast adhesion, proliferation and differentiation, in addition to improved mechanical performance [8]. Preliminary clinical and animal research has indicated that bone-to-implant contact values with Ti-Zr alloys were similar or superior to those with conventional titanium implants, indicating that the biological response of Ti-Zr alloys is very promising [9]. Clinically, titanium-zirconium implants may offer the following potential benefits reduced implant diameters, no significant loss of strength, enhanced resistance to fatigue fracture and improved distribution of occlusal forces [10, 11]. Therefore, it is of interest to compare titanium-zirconium alloy implants with traditional titanium implants in terms of clinical stability, peri-implant tissue response and overall success rate.

## Materials and Methodology:

## Study design and setting:

The proposed randomized clinical trial was done in the Department of Oral and Maxillofacial Surgery/Implantology of a tertiary dental health institution after securing Institutional Ethics Committee approval. All participants provided written informed consent before being enrolled in the study.

## Population and sampling of the study:

The study involved 40 patients who needed dental implants, either one or more, to replace the missing teeth. The sample was divided randomly into two groups:

[1] Group I (Test Group): This group of patients was receiving titanium-zirconium alloy implants.

[2] Group II (Control Group): Patients who have commercially pure titanium implants.

The randomization was performed using a computer-generated allocation sequence. Uniform allocation of implants across groups was performed.

## Inclusion criteria:

[1] Patients between 18 and 65 years old who need implant-supported prosthetic rehabilitation.

[2] The height and width of bones are sufficient to implant without major grafting.

[3] Proper oral health and follow-up incentive.

[4] No current periodontal disease.

## Exclusion criteria:

[1] Uncontrolled systemic diseases, e.g., diabetes mellitus or immunocompromised conditions in patients.

[2] Head and neck radiotherapy history.

[3] Heavy smokers (10 or more cigarettes/day)

[4] Pregnant or lactating women

[5] Bruxism patients who have parafunctional habits.

## Implant materials:

The experiment used commercially procured implants with the same macro-design and surface treatment and differentiated only in terms of material composition:

[1] Alloy implants made of titanium-zirconium alloys (Ti -Zr)

[2] Titanium implants commercially pure.

[3] No design bias was introduced by the implants' similarity in diameter and length.

## Surgical procedure:

The process of implant placement received local anesthesia and was performed in accordance with the standard aseptic surgical procedure. A mid-crestal incision was provided and full-thickness mucoperiosteal flaps were raised. Sequential drilling was used to prepare the osteotomy as recommended by the manufacturer. Controlled insertion torque was used to place implants and primary stability was measured. Depending on the selected healing protocol, cover screws/healing abutments were used. The position of flaps was repositioned and sutures were performed with interrupted sutures. The treatment was performed postoperatively using antibiotics, analgesics and a chlorhexidine mouth rinse. Sutures were removed in 7-10 days.

## Prosthetic phase:

Second-stage surgery was then performed when and where required, following a 3-4 months period of mandibular and a 4-6 months period of maxillary healing abutments. Conclusive rehabilitation was performed using screw-retained or cement-retained prostheses.

## Parameters of clinical evaluation:

Baseline, implant placement and prosthetic loading were used to determine the patients and periodic follow-ups (1 month, 3 months, 6 months and 12 months) were conducted. The parameters that were recorded are the following:

Primary implant stability (insertion torque /resonance frequency analysis, where applicable)

[1] Peri-implant soft tissue (gingival index, bleeding on probing)

[2] Probing depth by the implants.

## Availability of pain, infection, or mobility:

[1] Radiographic marginal bone loss measured with standardized intraoral periapical radiographs.

[2] Success and survival of implants.

## Outcome measures:

The first outcome was 1-year survival and success in the implant. Marginal bone loss, peri-implant tissue health and mechanical complications were the secondary outcomes.

## Statistical analysis:

The data were entered into Microsoft Excel and analyzed using statistical software. The quantitative variables were reported as mean and standard deviation and the qualitative variables as percentages. The independent t-test for continuous variables and the chi-square test for categorical variables were used to conduct intergroup comparisons. A p-value below 0.05 was considered statistically significant.

## Results:

A total of 40 patients with 40 implants were included in the study, with 20 implants placed in the titanium-zirconium (Ti-Zr) group and 20 in the commercially pure titanium group. All patients completed the 12-month follow-up period. No patients were lost to follow-up. The analysis compared implant stability, peri-implant tissue response, marginal bone loss and survival rates between the two groups. The demographic distribution of patients and implant sites was comparable between the two groups. No statistically significant difference was observed in gender distribution or implant location (p>0.05), indicating that the two groups were well matched at baseline (Table 1 - see PDF). At the end of 12 months, Ti-Zr implants demonstrated slightly better clinical outcomes, including higher primary stability and healthier peri-implant tissues. Although these differences favored the Ti-Zr group, they did not reach statistical significance (p>0.05). Implant mobility was observed only in the titanium group (Table 2 - see PDF). Radiographic evaluation revealed that a significantly higher proportion of Ti-Zr implants exhibited minimal marginal bone loss (<1 mm) compared to titanium implants (85% versus 65%, p=0.04). Although implant survival was slightly higher in the Ti-Zr group, the difference did not reach statistical significance (Table 3 - see PDF).

## Discussion:

The current clinical trial compared titanium-zirconium (Ti-Zr) alloy implants with traditional commercially pure titanium implants based on implant stability, peri-implant tissue response, marginal bone preservation and survival rate. The results of this research indicate that although the two implant materials reported high success rates, Ti-Zr implants exhibited greater marginal bone preservation and a tendency toward better clinical stability [3]. Titanium has been considered the standard implant material since the 1980s, due to findings of osseointegration and long-term survival rates of 90 to 96 percent in most clinical trials. The survival rate of titanium implants in the current study was 90 per cent, which is consistent with other published studies, indicating that the survival rate is 88-95 per cent at the same follow-up period. Compared with the Ti-Zr group, the Ti-Zr group exhibited 100 percent survival, which was reinforced by previous clinical findings demonstrating that alloy modification could enhance mechanical resistance and minimise the probability of implant failure [12, 13]. Marginal bone preservation has been regarded as one of the most relevant signs of implant success. In the current study, 85 percent of Ti-Zr implants showed marginal bone loss of less than 1 mm, compared with 65 percent in titanium, a statistically significant difference. The same results have been indicated in multicenter trials of implants, where Ti-Zr implants showed an average of 1520% less crestal bone loss than pure titanium implants in the first year of loading [14]. Enhanced bone retention in the region of Ti Zr implants could also be explained by the fact that these implants have greater mechanical strength and better force distribution under occlusal loading, thus decreasing stress concentration on the crestal bone [15]. Another important factor affecting osseointegration is the primary stability of the implants. The primary stability of Ti-Zr implants was good in 95 percent of cases, compared with 85 percent in the titanium group. This observation is consistent with biomechanical research indicating that Ti-Zr alloys have tensile strengths up to 30-40 percent higher than pure titanium. Stronger implants permit smaller implant diameters to be used without loss of stability, which could explain why the alloy category of implants is found to have higher stability levels [7, 16]. There were also positive trends in peri- implant soft tissue health in the Ti-Zr group. The results showed that healthy peri-implant mucosa was found in 90 percent of Ti-Zr implants and 75 percent of titanium implants; bleeding on probing was found in 10 percent of Ti-Zr implants and 25 percent of titanium implants. The current results align with clinical reports that Ti-Zr alloys with enhanced implant surface properties can enhance soft tissue adaptation and minimize inflammatory reactions. Ti-Zr surfaces have also been shown to promote osteoblast adhesion and proliferation in experimental cell-culture models, which could contribute to better biological reemergence [17]. The mechanical excellence of Ti-Zr alloys can be especially beneficial in demanding clinical procedures, such as thin ridges or posterior areas with deep cavities. Fracture rates of narrow titanium implants have been reported as 1-3 per cent in clinical implant registries; Ti-Zr implants have a lower fracture rate, of less than 1 per cent. Although this study did not find any implant fracture, the enhanced mechanical profile of Ti-Zr implants may be used in locations with limited bone volume, where conventional implants are prone to mechanical failure [14]. In general, the findings of the current study are consistent with emerging evidence that Ti-Zr implants have the same capacity for osseointegration and better mechanical characteristics. Although the clinical success of both implant types was high, marginal bone preservation in the Ti-Zr group was significantly lower, suggesting a long-term benefit of alloy implants.

## Conclusion:

Both titanium-zirconium and conventional titanium implants demonstrated high survival rates with favorable peri-implant tissue responses in this clinical trial. Ti-Zr implants showed superior marginal bone preservation, enhanced primary stability and better soft tissue health, particularly in mechanically demanding cases. Thus, Ti-Zr alloys emerge as a reliable alternative to traditional titanium implants when high strength or reduced diameters are clinically required.
